# Patients with Invasive Lobular Carcinoma Show a Significant Increase in IRS-4 Expression Compared to Infiltrative Ductal Carcinoma—A Histopathological Study

**DOI:** 10.3390/medicina58060722

**Published:** 2022-05-28

**Authors:** Miguel A. Ortega, Oscar Fraile-Martinez, Cielo García-Montero, Sandra Borja-Vergel, Diego Torres-Carranza, Leonel Pekarek, Coral Bravo Arribas, Juan A. De León-Luis, Cristina Sánchez-Rojo, Miguel Angel Alvarez-Mon, Natalio García-Honduvilla, Julia Buján, Santiago Coca, Melchor Alvarez-Mon, Miguel A. Saez, Luis G. Guijarro

**Affiliations:** 1 Department of Medicine and Medical Specialities, Faculty of Medicine and Health Sciences, University of Alcalá, 28801 Alcala de Henares, Spain; oscarfra.7@hotmail.com (O.F.-M.); cielo.gmontero@gmail.com (C.G.-M.); sandraborjavergel@gmail.com (S.B.-V.); diegotc90@gmail.com (D.T.-C.); leonel.pekarek@gmail.com (L.P.); maalvarezdemon@icloud.com (M.A.A.-M.); natalio.garcia@uah.es (N.G.-H.); mjulia.bujan@uah.es (J.B.); s.coca@uah.es (S.C.); mademons@gmail.com (M.A.-M.); 2 Ramón y Cajal Institute of Sanitary Research (IRYCIS), 28034 Madrid, Spain; luis.gonzalez@uah.es; 3 Cancer Registry and Pathology Department, Principe de Asturias University Hospital, 28806 Alcala de Henares, Spain; 4 Oncology Service, Guadalajara University Hospital, 19002 Guadalajara, Spain; 5 Department of Obstetrics and Gynecology, University Hospital Gregorio Marañón, 28009 Madrid, Spain; cbravoarribas@gmail.com (C.B.A.); jaleon@ucm.es (J.A.D.L.-L.); 6 Health Research Institute Gregorio Marañón, 28009 Madrid, Spain; 7 Department of Obstetrics and Gynecology, Central University Hospital of Defence-UAH Madrid, 28801 Alcala de Henares, Spain; cristina.sanchez.rojo@gmail.com; 8 Immune System Diseases-Rheumatology, Oncology Service an Internal Medicine, University Hospital Príncipe de Asturias, 28806 Alcala de Henares, Spain; 9 Pathological Anatomy Service, Central University Hospital of Defence-UAH Madrid, 28801 Alcala de Henares, Spain; 10 Unit of Biochemistry and Molecular Biology, Department of System Biology, University of Alcalá, 28801 Alcala de Henares, Spain

**Keywords:** breast cancer (BC), invasive lobular carcinoma (ILC), insulin receptor substrate 4 (IRS-4), cyclooxygenase 2 (COX-2), cyclin D1, retinoblastoma protein 1 (Rb1)

## Abstract

*Background and Objectives:* Breast cancer (BC) is the first diagnosed type of cancer and the second leading cause of cancer-related mortality in women. In addition, despite the improvement in treatment and survival in these patients, the global prevalence and incidence of this cancer are rising, and its mortality may be different according to the histological subtype. Invasive lobular carcinoma (ILC) is less common but entails a poorer prognosis than infiltrative ductal carcinoma (IDC), exhibiting a different clinical and histopathological profile. Deepening study on the molecular profile of both types of cancer may be of great aid to understand the carcinogenesis and progression of BC. In this sense, the aim of the present study was to explore the histological expression of Insulin receptor substrate 4 (IRS-4), cyclooxygenase 2 (COX-2), Cyclin D1 and retinoblastoma protein 1 (Rb1) in patients with ILC and IDC. *Patients and Methods:* Thus, breast tissue samples from 45 patients with ILC and from 45 subjects with IDC were analyzed in our study. *Results:* Interestingly, we observed that IRS-4, COX-2, Rb1 and Cyclin D1 were overexpressed in patients with ILC in comparison to IDC. *Conclusions:* These results may indicate a differential molecular profile between both types of tumors, which may explain the clinical differences among ILC and IDC. Further studies are warranted in order to shed light onto the molecular and translational implications of these components, also aiding to develop a possible targeted therapy to improve the clinical management of these patients.

## 1. Introduction

Breast cancer (BC) is the most common type of diagnosed cancer in women and the second leading cause of cancer-related mortality in this group [1,2]. Following the American Cancer Society, 1 in 8 women will suffer from BC in their lives, and it has been projected that the global incidence of these tumors will reach 3.2 million new cases per year by 2050 [3]. Moreover, men can also suffer from BC, accounting for less than 1% of all cancers in men and less than 1% of all breast cancers for them [4]. Lifestyle habits like smoking, sedentarism, alcohol consumption and diet are some of the most important risk factors for suffering from BC, along with obesity, aging, race, hormonal/reproductive factors and history of familiar BC [5]. An early diagnosis and regular screening are crucial for a good prognosis and survival rate of BC patients. Indeed, thanks to these approaches, the 5-year relative survival rate can be over 80% in some developed countries [6]. However, the overall survival of these patients will depend mainly on two factors: the tumoral stage (invasiveness, metastasis), and the tumoral subtype [7]. According to its histological classification, the most common type of invasive BC is the infiltrative ductal carcinoma (IDC) of no special subtype [8]. Approximately 1 in 4 tumors are defined as histological ‘special types’, including at least 17 discrete pathological entities such as invasive lobular carcinoma (ILC) [9]. Furthermore, ILC is the second most common subtype of BC, and differs from IDC in a set of clinical and histopathological features. For instance, ILCs present more difficulties in their detection, often exhibit a poorer prognosis, more advanced stages, frequent late recurrences and lower responses to therapy [10]. In addition, there are a plethora of biological differences between these types of tumors, including in their molecular profiles, immune response, metastasis, metabolism and other hallmarks of cancer [11,12]. Thus, it is necessary to deepen examinations on the molecular and biological basis of ILC in order to develop further approaches and strategies to aid in the clinical management of this relevant malignancy.

Insulin receptor substrate 4 (IRS-4) is a relevant molecule altered in different types of malignancies like BC. It seems that this molecule promotes tumoral proliferation and therapy resistance due to the alteration of different molecular pathways [13,14,15]. Furthermore, previous studies have found that an overexpression of this component is frequently related to a poorer prognosis [16]. A similar role has been found with cyclooxygenase 2 (COX-2) in patients with BC, being frequently related to a poorer prognosis and a set of carcinogenic mechanisms [17]. In the same line, Cyclin D1 levels are often disrupted in different types of tumors, especially in BC, where approximately 50% of mammary carcinomas present an overexpression of this component [18]. Likewise, the retinoblastoma protein 1 (Rb1) is also involved in the tumorigenesis and impaired cell features, having been proposed as a promising therapeutic target of different types of tumors, including BC [18,19]. Despite the relevance and demonstrated role of these markers in the development and progression of BC, there is little evidence collected regarding the differential expression of each component in ILC vs. IDC. 

Hence, the aim of the present review is to explore the histopathological detection of IRS-4, COX-2, Rb1 and Cyclin D1 in 45 patients with ILC in comparison to 45 IDC in order to establish potential biological differences between both types of tumors. With that purpose, we have conducted immunohistochemical studies of n patients with ILC and compared these with n patients with IDC. 

## 2. Patients and Methods

### 2.1. Collection of Samples

For our study, we used paraffin-embedded sections of breast tissue from 45 patients diagnosed with invasive lobular carcinoma (ILC) and 45 patients diagnosed with infiltrative ductal carcinoma (IDC). The diagnosis followed the principles of Lakhani et al. [20]. The present study was designed as an observational, analytical, retrospective cohort study with longitudinal follow-up. Paraffin blocks and all details with extensive clinical information on patients and follow-up data were retrospectively reviewed.

The study was carried out in accordance with the basic ethical principles of autonomy, beneficence, non-maleficence and distributive justice, and its development followed the rules of Good Clinical Practice, the principles contained in the most recent Declaration of Helsinki (2013) and the Convention of Oviedo (1997). The data and information collected complied with current legislation on data protection (Organic Law 3/2018 of 5 December on the Protection of Personal Data and Guarantee of Digital Rights and Regulation (EU) 2016/679).

### 2.2. Histopathological and Immunohistochemical Studies

Immunohistochemical studies were performed on paraffin-embedded breast tissue samples. The antibody recovery step was described in the protocol specifications (Table 1). Antigen/antibody reactions were detected by the avidin-biotin complex (ABC) method, with avidin-peroxidase, following the protocols of Ortega et al. [21]. After incubation with the primary antibody (1 h and 30 min), samples were incubated with 3% BSA blocker (catalog #37525; Thermo Fisher Scientific, Inc., Waltham, MA, USA) and PBS overnight at 4 °C. The samples were then incubated with biotin-conjugated secondary antibody and diluted in PBS for 90 min at room temperature (RT; Rabbit IgG (RG-96, 1:1000, Sigma-Aldrich/Mouse IgG (F2012/045K6072) 1:300, Sigma-Aldrich, St. Louis, MI, USA). Avidin-peroxidase conjugate ExtrAvidin^®^-Peroxidase (Sigma-Aldrich; Merck KGaA, Darmstadt, Germany) was used for 60 min at RT (1:200 dilution with PBS), and then the level of protein expression was determined using a Chromogenic Diaminobenzidine (DAB) Substrate Kit (cat. no. SK-4100; Maravai LifeSciences, San Diego, CA, USA), which was prepared immediately prior to exposure (5 mL distilled water, two drops of buffer, four drops of DAB and two drops of hydrogen peroxide drops). The signal was developed with the chromogenic peroxidase substrate for 15 min at RT; this technique allows the detection of a brown staining. For the detection of each protein, sections of the same tissue were assigned as negative controls, substituting the incubation with the primary antibody for a blocking solution (PBS). In all tissues, contrast was performed with Carazzi hematoxylin for 15 min at RT.

### 2.3. Histopathological Evaluation

Tissue sections were viewed using a Zeiss Axiophot light microscope (Carl Zeiss, Oberkochen, Germany) equipped with an AxioCam HRc digital camera (Carl Zeiss, Oberkochen, Germany). Given the important role of the proteins studied, the evaluation of the histological results was carried out according to the intensity of expression for the immunohistochemical staining with Score. Therefore, the histological samples of patients diagnosed with breast cancer were classified as negative (0) or low/medium (1) and high (3) expression using the IRS-Score method [22]; the samples were evaluated by two independent pathologists (MAO, MAS), and in case of discrepancies, a third pathologist intervened (SC). For each established subject group, seven randomly selected microscopy fields were examined in each of the five sections. Subjects were classified as positive when the mean proportion of the labeled sample was greater than or equal to 5% of the total sample. This was completed by calculating the total percentage of the labeled tissue in each microscopy field to obtain a mean for the study sample as described [23]. The observation and quantification of the samples were carried out independently by two researchers.

### 2.4. Statistic Analysis

For the statistical analysis, the statistical package GraphPad Prism^®^ 5.1 was used for the Mann–Whitney U test as appropriate. The data are provided as the mean ± standard deviation (SD). The error bars in the figures indicate the SD. Different levels of significance are distinguished as * *p* < 0.05, ** *p* < 0.005 and *** *p* < 0.001.

## 3. Results

### 3.1. Clinical and Sociodemographic Characteristics of the Study Population

The present study was designed as an observational, analytical, retrospective cohort study with longitudinal follow-up. A total of 45 patients with ILC and 45 patients with IDC were analyzed, with a median age of 69.167 ± 13.663 years for ILC and 67.571 ± 10.717 years for IDC. All patients had a score greater than pT1. The percentage of expression of estrogen receptors was 70.000 ± 22.887% for ILC and 66.538 ± 28.091% for IDC. The percentage of expression of progesterone receptors was 54.333 ± 26.245% for ILC and 55.833 ± 33.086% for IDC. The Ki67 expression percentage was 12.609 ± 6.373% for ILC and 16.190 ± 7.731% for IDC.

### 3.2. Patients with Invasive Lobular Carcinoma Show a Significant Increase in the Expression of IRS-4

**Figure 1:**
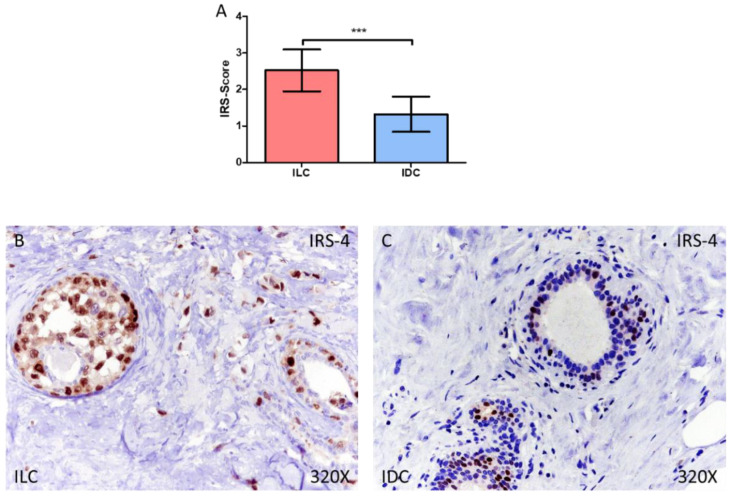
(**A**) IRS-Score for IRS-4 in patients diagnosed with invasive lobular carcinoma (ILC) and infiltrative ductal carcinoma (IDC). (**B**,**C**) Histological images of IRS-4 expression using immunohistochemical techniques in the breast tissue of patients diagnosed with ILC (**B**) and IDC (**C**). *** *p* < 0.001.

Our results demonstrate how patients with ILC show an increased expression of IRS-4 in the tissue compared to IDC patients. We observed how the IRS-Score expression score was 2.522 ± 0.574 in ILC and 1.322 ± 0.479 in IDC, *** *p* < 0.001 (Figure 1A–C).

### 3.3. Patients with Invasive Lobular Carcinoma Show a Significant Increase in the Expression of C0X-2, Rb1 and Cyclin D1

**Figure 2:**
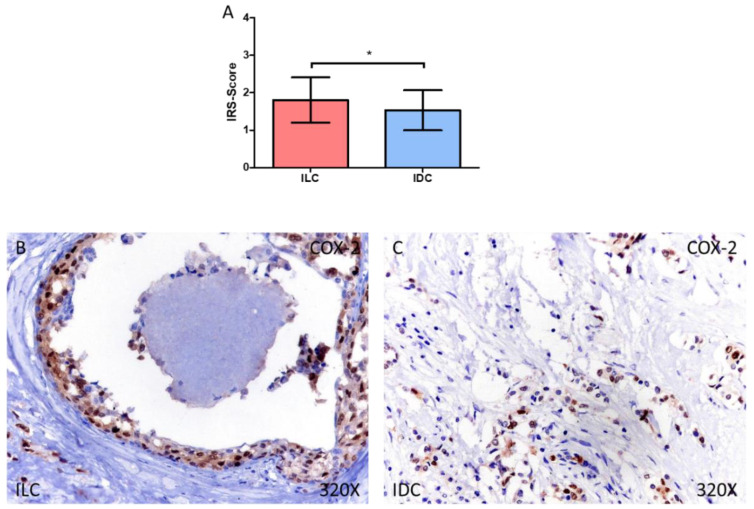
(**A**) IRS-Score for COX-2 in patients diagnosed with invasive lobular carcinoma (ILC) and infiltrative ductal carcinoma (IDC). (**B**,**C**) Histological images of COX-2 expression using immunohistochemical techniques in the breast tissue of patients diagnosed with ILC (**B**) and IDC (**C**). * *p* < 0.05.

In addition, we have observed how patients diagnosed with ILC show an increased expression of COX-2, Rb1 and Cyclin D1. In the case of COX-2, we observed how the IRS-Score expression score was 1.806 ± 0.605 in ILC and 1.533 ± 0.537 in IDC, * *p* = 0.0267 (Figure 2A–C).

**Figure 3:**
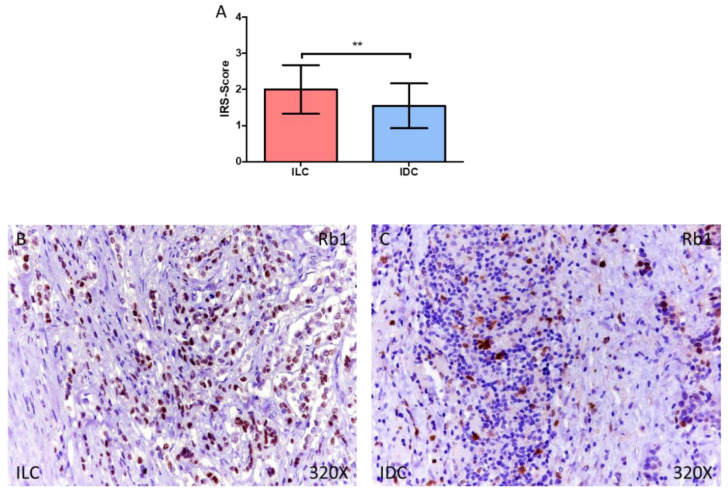
(**A**) IRS-Score for Rb1 in patients diagnosed with invasive lobular carcinoma (ILC) and infiltrative ductal carcinoma (IDC). (**B**,**C**) Histological images of Rb1 expression using immunohistochemical techniques in the breast tissue of patients diagnosed with ILC (**B**) and IDC (**C**). ** *p* < 0.005.

**Figure 4:**
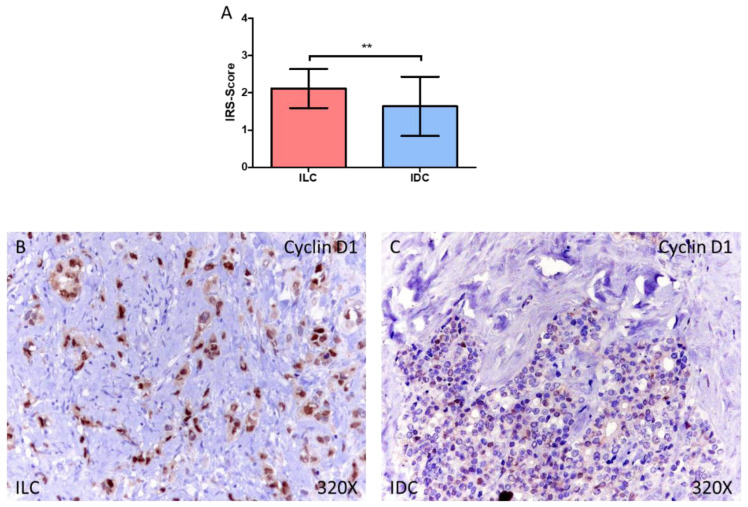
(**A**) IRS-Score for Cyclin D1 in patients diagnosed with invasive lobular carcinoma (ILC) and infiltrative ductal carcinoma (IDC). (**B**,**C**) Histological images of Cyclin D1 expression using immunohistochemical techniques in the breast tissue of patients diagnosed with ILC (**B**) and IDC (**C**). ** *p* < 0.005.

For Rb1, we observed how the IRS-Score expression score was 2.000 ± 0.674 in ILC and 1.544 ± 0.620 in IDC, ** *p* = 0.0022 (Figure 3A–C). In the case of Cyclin D1, we observed how the IRS-Score expression score was 2.117 ± 0.527 in ILC and 1.639 ± 0.793 in IDC, ** *p* = 0.0018 (Figure 4A–C).

## 4. Discussion

The annual incidence and prevalence of BC are rising worldwide in the younger and elderly populations [24]. Despite the improvements achieved in early diagnosis, screening and survival, more knowledge is required to understand the biology of this cancer and their specific types in order to develop better approaches for the clinical management of these patients. In this sense, our study gains further insights into the biology of ILC in comparison to IDC, aiding to explain some of the differences in the clinical presentation and histopathological features of these types of tumors. 

ILCs are frequently associated with a poorer prognosis than IDCs, and the biological differences between both types of tumors have received significant attention. For instance, differences in the expression of genes and specific proteins/receptors have been reported among both groups [25]. IRS-4 was a pivotal marker overexpressed in ILC in comparison to IDC. To our knowledge, this is the first study demonstrating a possible pathophysiological role of this marker in these tumors. IRS-4 is an adaptor protein acting as a constitutive activator of critical cell transduction pathways in cancer, leading to the activation of the PI3K/Akt pathway collaborating with the actions of the human epidermal growth factor receptor 2 (HER2) [13]. HER2 is a central receptor involved in BC development and stratification. According to the absence or presence of HER2, estrogen receptor (ER) and progesterone receptor (PR), the molecular classification of breast tumors can be luminal A, luminal B, HER2+ enriched cells and triple negative BC. Luminal subtypes are ER/PR+, being luminal A subtype negative for HER2 and with low levels of ki67, whereas luminal B presents the HER2+ receptor and high levels of ki67. HER2+ enriched cells only present this marker, and triple negative BC lacks all of these receptors [26,27]. It is of note, that patients with ILC were HER2+, whereas IDC subjects did not show HER2+ expression. HER2+ ILC has distinct clinical characteristics and immune landscapes compared to IDC, and a poorer prognosis [28]. Hence, the augmentation of both IRS-4 and HER2+ may act synergically with the progression of ILC. Moreover, compelling evidence has established the central role of PI3K in the development of BC, being closely related to cell growth, proliferation, survival, motility, metastasis, metabolism and immune modulation [29]. In addition, previous studies have also found an association between the activation of PI3K/Akt and other pathways such as Ras-MAPK by IRS-4 with therapeutic resistance and tumor progression in lung cancer [13]. Thus, IRS-4 could mediate many of these processes in ILCs due to its hyperactivation of PI3K/Akt and other signaling pathways, aiding to explain the worse prognosis of these patients in comparison to IDC. 

Likewise, the expression of IRS-4 was also related to the activation of Cyclin D1 and Rb1 in colorectal cancer, two markers also altered in our study [30]. Because of the increased expression of these components, it is likely that IRS-4 may collaborate with the overexpression of Cyclin D1 and Rb1. Cyclin D1 and Rb1 are major regulators of the cell cycle. In the case of Cyclin D1, prior research has detected a substantial dysregulation of this marker in several types of cancer [31]. In the field of BC, the overexpression of Cyclin D1 is associated with abnormalities in the cell cycle and a set of carcinogenic mechanisms in the breast, also mediating the effects of estrogen in this tissue [32]. Soslow et al. [33] claimed that 82% (23 out of 28) of ILCs exhibited a high expression of Cyclin D1 in comparison to the 54% of IDCs (18 out of 34). In agreement with previous studies, we have observed an increased expression of Cyclin D1 in ILCs in comparison to IDCs. In the case of Rb1, it may act as either a tumor suppressor or as promoting tumor growth [34]. It seems that the high expression of Rb1 is associated with a high proliferation of different invasive breast tumors [35]. Interestingly, the levels of Rb1 appear to be correlated with those of glucose transporter 1 (GLUT-1) in BC, suggesting a promising therapeutic approach using GLUT-1 inhibitors in patients with high Rb1 expression [36]. However, more studies are required to better understand the role of Cyclin D1 and Rb1 in lobular carcinoma before drawing any conclusions. 

Last but not least, COX-2 is an enzyme ubiquitously involved in the mammary carcinogenesis. Its expression appears to be directly correlated with the stage, cancer progression, angiogenesis and metastasis [37]. Furthermore, COX-2-driven prostaglandin E2 (PGE-2) biosynthesis is related to a plethora of aggressive carcinogenic mechanisms in BC, having been proposed as a promising therapeutic target and also being associated with a poorer prognosis [17,38]. COX-2 can be released by cancer-associated fibroblasts, M2 macrophages and cancer cells to the tumor microenvironment, inducing cancer stem cell-like activity and cell proliferation, angiogenesis, invasion, inflammation, metastasis and apoptotic resistance [39]. In consonance with our results, Holmes et al. [40] found that higher COX-2 expression was more observed in ILC than IDC, aiding to explain its worse prognosis. Other studies, however, found that COX-2 was highly expressed in both ILC and IDC, and the proportion of total COX-2 positive tumors range between studies [37,41]. These differences could be attributed to the different scoring systems and cut-off of immunohistochemistry, as these do not allow for the extraction of quantitative results, which may be a limitation of our study [42]. Similarly, COX-2 was also highly detected in lobular and ductal in situ carcinoma, being associated with an increased risk for developing subsequent invasive carcinomas [42,43], hence supporting the role of COX-2 in the early carcinogenesis as well. 

## 5. Conclusions

Overall, we have found a differential molecular profile between ILC and IDC, showing an increased expression of IRS-4, COX-2, Cyclin D1 and Rb1 in favor of the former. Further studies are warranted in order to deepen exploration on the molecular and translational implications of these components, as well as to analyze a possible targeted therapy to improve the clinical management of these patients. 

## Figures and Tables

**Table 1:** Primary antibodies used, together with the dilutions and protocol specifications.

Antigen	Dilution	Provider	Protocol Specifications
IRS-4	1:250	Thermo Fisher Scientific—PA5-117329	Preincubation with Tris-EDTA buffer pH 9 and incubation with 0.1% TTX (Triton ×100 in TBS) for 5 min.
COX-2	1:750	Vitro, MAD-000335QD-3/V	-----------------
Rb1	1:500	Vitro, MAD-000900QD-3/V	-----------------
Cyclin D1	1:500	Vitro, MAD-000630QD-3/V	Preincubation with Tris-EDTA buffer pH 9 and incubation with 0.1% TTX (Triton ×100 in TBS) for 5 min.

## Data Availability

The datasets used and/or analyzed during the present study are available from the corresponding author on reasonable request.
